# Tests for the Detection of Venereal Diseases

**Published:** 1913-01

**Authors:** 


### Tests for the Detection of Venereal Diseases.

APROPOS TO REQUIRING A HEALTH CERTIFICATE FOR MARRIAGE.

Public sentiment is rapidly swinging around to the viewpoint of requiring a health certificate before a marriage license is issued, showing the parties to be free from any venereal disease or condition that would affect the propagation of a normal child. The recent exposure of the widespread prevalence of venereal diseases and the suffering and wreckage following their wake has put good men and women to more than mere thinking. There is a decided conviction that this shame on our civilization must and shall be corrected. Heretofore ignorance and the *mores* of silence touching any question sexual has precluded any advancement in education along this line, and that great bugbear, "personal liberty," has maintained sufficient prestige to checkmate any legislation regulating the question of marriage. But, fortunately for the future of the race, the forces that be, especially the interest in eugenics, has paved the way for a new era in education. Already several States have passed restrictive laws safeguarding the home and posterity. But it must not be inferred that by the term "restrictive" it is meant that the individual is deprived of the fullest privileges of exercising his right of sexual selection. It is but a preventive measure protecting the home from a scourge infinitely worse and more far-reaching than any infectious disease which the State now exercises the right to control. Its relative importance is as great, when applied to the uplift of man, as that which concerns the spiritual in comparison with the physical.

Now that the public has been taught that syphilis extends its baneful effect to the third and fourth generation, and that gonorrhea is seldom ever completely cured in the majority of cases, there very pertinently arises the question: By what means can the facts be ascertained that any person is free from conveying either contagion? It is only in the last year or two that we have had at our command any very certain means to determine this matter, and it is this knowledge that gives a practical application to the positive side of eugenics.

For the special instruction of physicians and others interested in the problems of eugenics, we give here a most valuable paper by Dr. Williams, "Some Notes on the Laboratory Side of Eugenics." This paper will be followed by others in amplification of the serum test for gonorrhea and the Wassermann test for syphilis. D.

